# Can prematurity risk in twin pregnancies after in vitro fertilization be predicted? A retrospective study

**DOI:** 10.1186/1477-7827-7-136

**Published:** 2009-11-25

**Authors:** Andrea Weghofer, Katharina Klein, Maria Stammler-Safar, Christof Worda, David H Barad, Peter Husslein, Norbert Gleicher

**Affiliations:** 1 Department of Obstetrics & Gynecology, Medical University Vienna, Vienna, Austria; 2 The Center for Human Reproduction and The Foundation for Reproductive Medicine, New York, New York, USA; 3 Department of Obstetrics, Gynecology and Reproductive Sciences, Yale Universiy School of Medicine, New Haven, Connecticut, USA

## Abstract

**Background:**

Assisted reproduction (ART) contributes to world-wide increases of twin pregnancies, in turn raising prematurity risks. Whether characteristics of ART cycles, resulting in twin gestations, can predict prematurity risks was the subject of this study.

**Methods:**

One-hundred-and-six women, ages 20 to 39 years, with consecutive dichorionic-diamniotic (DC/DA) twin gestations were retrospectively investigated. All pregnancies investigated followed fresh ART cycles, with use of autologous gamets, and were delivered at a university-based high-risk, maternal-fetal medicine unit. Only premature deliveries (i.e., <37.0 weeks gestational age), with viable neonate(s) of ≥ 500 grams, were considered for analysis.

**Results:**

After 1.8 +/- 1.2 ART cycles, 11.0 +/- 5.4 oocytes were retrieved and 2.4 +/- 0.9 embryos transferred in 106 women aged 31.6 +/- 4.2 years. Indications for ART treatment were male factor in 51.9%, female infertility in 27.4% and combined infertility in 20.8%. Though maternal age significantly influenced prematurity risk (p < 0.05), paternal age, maternal body mass index, indications for fertility treatment, number of previous ART attempts, oocytes retrieved or embryos transferred, as well as stimulation protocols and previous ART pregnancies, were not associated with gestational duration in twin pregnancies.

**Summary:**

Except for female age, baseline and ART cycle characteristics do not allow for prediction of prematurity risk in dichorionic twin gestations after assisted reproduction.

## Background

Since Louise Brown, the first in vitro fertilization (IVF) birth in 1978, more than three million IVF children have been born [1]. Assisted reproduction (ART), nevertheless, has come under criticism due to the increased risk of multiple births associated with this fertility treatments [2]. Twin pregnancies after spontaneous conception occur in Caucasians at a rate of 1:80. Assisted reproduction in Europe, in contrast, currently results in twin rates of 21.7% with wide variations across the countries [3]. Twins (and higher order multiples), in comparison to singleton gestations, are at increased risk for prematurity-associated adverse perinatal outcome [4,5]. A broader application of single embryo transfer (SET) has been suggested to reduce this rising incidence of multiple pregnancies in the course of assisted reproduction without compromising pregnancy chances [6,7].

Bechoua et al., for instance, describe comparable pregnancy chances after double embryo transfer (DET) when compared to elective single embryo transfer in a cohort of good prognosis patients [8]. A Finnish group even report lower costs and higher cumulative live birth rates in good prognosis patients after SET and cryo-embryo transfers compared to DET [9,10]. Others, however, claim that SET may after all reduce pregnancy chances. Veleva et al., for instance, differentiate between elective and compulsory SET. In their retrospective analysis, they describe considerably lower pregnancy rates in women undergoing SET of a non top quality embryo or compulsory SET in comparison to double embryo transfers or elective SETs of a top quality embryo [11]. Roberts et al. go even further - they report a need for a 55% rate of SET to reduce twin rates to ten percent. By doing so, pregnancy rates would drop by 19% [12]. In a recent meta-analysis, Gelbaya et al. are in accordance with these data. They report a statistically significant reduction in the probability of live birth (-38%) and multiple birth (-94%) after e-SET [13].

The ability to prospectively identify IVF patients at increased risk towards twinning and premature delivery, would, therefore, allow for a more selective utilization of SET and therefore, possibly, more effectively contribute to the reduction of adverse perinatal outcome in IVF twins [12]. Identifying such a subset of patients at risk, would then more than compensate for potentially impaired pregnancy chances in women who are not good prognosis candidates for SET.

While a variety of *prenatal *risk factors for preterm births in twin gestations have been established [14], conflicting data exist on the potential impact of controlled ovarian hyperstimulation on prematurity risk and perinatal outcome. Griesinger and colleagues, for instance, fail to find an impact of controlled ovarian hyperstimulation on birth weight [15], while Shih et al. report lower birth weight in singletons after fresh embryo transfer when compared to frozen cryopreservation cycles [16]. Abramov et al. go even further, showing higher rates of prematurity, low birth weight, pregnancy-induced hypertension and placental abruption in pregnancies after ovarian hyperstimulation syndrome (OHSS) [17]. Aytoz concur with these observations, describing increased rates of intrauterine deaths in ART pregnancies with severe male factor infertility [18].

These publications point toward a potentially negative influence of IVF cycle characteristics, such as male factor infertility [18] and good response to controlled ovarian hyperstimulation or OHSS [17], on pregnancy outcome and prematurity risk. Male factor infertility and OHSS are, however, considered risk factors for the occurrence of multiple gestations [12,19].

Whether other ART cycle characteristics, such as indication for fertility treatment and ovarian response to stimulation, serve as predictive for severe prematurity in twin gestations after ART was the subject of the here presented study.

## Discussion

### Methods

The present retrospective study involved 106 women, aged 20 to 39 years, with dichorionic-diamniotic (DC/DA) twin gestations after in vitro fertilization (IVF). All pregnancies were established with autologous oocytes/sperm in fresh IVF cycles and via the transfer of two or more embryos. To ensure IVF-related conception and a complete history of IVF-related parameters, such as the number of embryos transferred, only *IVF-Fonds-*covered pregnancies were included. [The *IVF-Fonds *provides governmental financial support of up to four IVF cycles per pregnancy in women under age 40 [20]]. To determine chorionicity, patients underwent first trimester ultrasound scans, performed by a small group of experienced, specifically and uniformly trained senior physicians. The detection of lambda signs served as proof of dichorionicity.

All women underwent prenatal care and delivery at the *Department of Obstetrics and Gynecology *at the *Medical University Vienna*, a University-based hospital unit for high-risk maternal-fetal medicine. To exclude potential prematurity-associated biases, neither monochorionic twin gestations, nor pregnancies that had undergone selective fetal reduction or pregnancies with vanishing embryos were eligible for enrollment. After 37 weeks of gestation, a number of twin pregnancies were delivered electively. To exclude this potential bias, only twin gestations that were delivered preterm (i.e., gestational age < 37.0 weeks) were included in the study. A comparison of IVF cycle characteristics between study patients and DC twin gestations that delivered at term (i.e., ≥ 37 weeks; not eligible for enrollment) revealed no significant differences between the groups.

Statistical influences of maternal and paternal ages, numbers of previous IVF attempts and pregnancies, indications for fertility treatment, stimulation protocols, oocyte yields, fertilization procedure (IVF or ICSI), number of embryos fertilised/transferred, smoking status and body mass index on gestational ages were investigated. The data analyses of pregnancy- and delivery-related maternal and neonatal outcome data were based on retrospective chart reviews and computer-generated databases at the *Department of Obstetrics and Gynecology *at the *Medical University Vienna*. Assisted reproduction technology (ART)-related data were collected by chart review and, where applicable, from of a computer-generated database at the *IVF-Fonds*.

### Statistics

Statistical analyses were performed utilizing SPSS version 10.0. Quantitative variables are summarized by their mean (standard deviation), while qualitative variables are summarized by frequency tables. Univariate and multivariate analysis were performed by (stepwise) linear regression. Pearson's Correlation was used to determine the sample size required to detect a correlation between gestational duration and ART cycle characteristics. In order to detect a correlation of 0.25 with a power of 80% and a Type I error of 5%, a sample size of 98 women was needed. Patient characteristics known to influence prematurity risk in spontaneous pregnancies (i.e., age, smoking status, body mass index, parity) [14] were investigated in univariate analysis and included in multivariate analysis if significant (i.e., p < 0.05). ART cycle characteristics (i.e., numbers of previous IVF attempts and pregnancies, indications for fertility treatment, stimulation protocols, oocyte yields, fertilization procedure (IVF or ICSI), number of embryos fertilized/transferred) were also investigated in univariate regression analysis. While number of previous IVF-attempts, stimulation protocols, fertilization procedure (IVF or ICSI) and number of embryos transferred were only included in multi-regression analysis if significant in univariate analysis, factors previously described to be associated with prematurity risk, such as maternal age, number of previous IVF-pregnancies, indication for fertility treatment and oocyte yield [14,17,18], were included in multivariate regression analyses, irrespective of their statistical significance in univariate analysis. Univariate and multivariate regression analyses were then performed in the same fashion for women ≤ 30 years and > 30 years, except for the exclusion of maternal age.

A comparison of ART cycle characteristics between study patients and DC twin gestations that delivered at term (i.e., ≥ 37 weeks; not eligible for enrollment) revealed no significant differences between the groups. P-values of < 0.05 were considered statistically significant. Institutional Review Board (IRB) approval for data-linkage and retrospective data analyses was obtained from the IRB at the *Medical University Vienna*.

## Results

Women demonstrated a mean age of 31.6 +/- 4.2 years and a mean body mass index of 23.7 +/- 4.34 kg/m^2^. Men presented with a mean age of 33.9 +/- 4.5 years. Indications for in vitro fertilization treatment were male factor in 51.6%, female infertility in 27.4% and combined infertility in 20.8%. Women with female infertility demonstrated with tubal infertility in 64.7%, 23.5% were patients with polycystic ovary syndrome (PCOS), 9.8% suffered from endometriosis and 2% had a history of tubal ligation. In addition to PCOS, one patient demonstrated with infertility-associated immunological factors. Due to the present distribution of fertility indications, intracytoplasmatic sperm injection (ICSI) was required in 68.9% of cases.

To achieve their dichorionic-diamniotic pregnancies, couples underwent a mean number of 1.8 +/- 1.2 ART cycles. More than two ART attempts were required in 21.7% of patients. IVF attempts with concurrent twin pregnancy demonstrated a mean number of 11.0 +/- 5.4 oocytes retrieved and 6.6 +/- 3.5 oocytes fertilised. A mean number of 2.4 +/- 0.9 embryos were transferred. No statistically significant difference was observed in the number of embryos transferred according to cycle number and previous IVF outcome (first IVF attempts, 2.3 +/- 0.7; previous IVF failure, 2.7 +/- 1.1; previous IVF pregnancies, 2.4 +/- 0.9 embryos). The couples' ages, their indications for fertility treatments, oocyte yields, fertilization rates and other IVF/ICSI cycle characteristics were also comparable between first ART attempts, previous ART failures and previous ART pregnancies.

Delivery occurred at a mean gestational age of 33.6 +/- 2.9 weeks and resulted in 105 female and 107 male neonates. The mean birth weight for both twins was 2011.3 +/- 527.9 gm (Twin A) and 1927.9 +/- 549.2 gm (Twin B), respectively, with a mean discordance of 9.6 +/- 13.4 percent. Mean perinatal arterial pH for both twins was 7.26 +/- 0.1 (Twin A) and 7.26 +/- 0.1 (Twin B). Delivery resulted in 99.1% live births for Twin A and B. Immediately post partum, 46.2% of first twins and 50.0% of second twins required neonatal intensive care at the neonatal intensive care unit (NICU) of the *Department of Pediatrics *at the *Medical University Vienna*.

When the impact of maternal and paternal age, maternal body mass index and tobacco usage, previous pregnancies and IVF attempts, stimulation (GnRH agonist or GnRH antagonist protocol), oocyte yield, fertilization rate and number of embryos transferred on gestational age were calculated in univariate analysis, only maternal age reached statistical significance (p < 0.05). In multi-regression analyses, including maternal age, oocyte yield and number of previous IVF-pregnancies, only maternal age demonstrated to be predictive for gestational duration.

When univariate and multivariate regression were performed according to age groups (i.e., ≤ 30 years and >30 years for female age), maternal age did not reach significance in univariate analysis and was, therefore, not included in multi-regression. When multi-regression analyses, including number of oocytes retrieved, number of previous IVF-pregnancies and indication for fertility treatment, were then performed for those age groups (i.e., ≤ 30 years and >30 years), none of those characteristics included proved statistically associated with prematurity risk in twin gestations.

Many improvements in perinatal and neonatal medicine have led to increased survival rates in premature neonates. Their neurodevelopmental outcomes, particularly of those with very low birth weight and/or very early gestational age, are, however, still concerning and often followed by a variety of lifelong disabilities [21]. Assisted reproduction represents such a risk factor for prematurity in both, singletons and multiple pregnancies [22].

The uniformly agreed to goal of ART is the safest possible delivery of greatly desired offspring. SET, in its current form, focuses, however, on prevention of twin pregnancies, rather than on reduction of risks, associated with twin pregnancies. By doing so, single embryo transfer is mainly performed in good prognosis, i.e. young, IVF patients [23]. Obstetrical data, however, demonstrate an exponentially increasing prematurity risk with advancing maternal age [24]. If we could prospectively identify a subset of women at increased risks after twin conception, following ART, those patients would especially benefit from single embryo transfer. Even women with impaired pregnancy chances after SET, such as older patients and those with diminished ovarian function [25,26], would derive compensatory benefits to lower pregnancy chances by a possible reduction in prematurity risk.

Such risk-focused, rather than age-based, treatment policies have already been applied in prenatal diagnosis. While amniocentesis used to be a routine procedure in women above age 35, this approach was abandoned in favor of an individual risk calculation [27]. This modification in approach increased diagnostic accuracy for fetal chromosomal abnormalities, but also saved a considerable number of prospective mothers of advanced age from the amniocentesis-related risks of a preterm premature rupture of membranes or miscarriage.

Our, unfortunately, negative findings in this study, may have a variety of explanations: (i) All women that were eligible for enrolment underwent close prenatal surveillance and delivery at a university-based high-risk, maternal-fetal medicine unit. Whatever ART-associated risk divergence may have been present, excellent prenatal care could have evened it out. (ii) This is further supported by IVF twins presenting with significantly lower perinatal mortality than spontaneously conceived twins [4,28]. (iii) Women in this study also do not necessarily reflect a typical IVF population. Our study patients represented IVF-patients under age 40 with normal body mass index; they were either themselves fertile (male factor) or suffered from tubal infertility, and had only a limited numbers of previous IVF attempts.

This stands in contrast to other indications for fertility treatments, which are statistically highly associated with impaired pregnancy outcome, such as autoimmunity, uterine abnormalities or a history of repeated pregnancy loss [29,30]. Moreover, our study population involved only fresh, autologous IVF cycles, reported associated with higher implantation potential and, consequently, higher risk for twin gestations, than cycles in which thawed embryos are used [31].

## Summary

Standard ART cycle characteristics did not serve as predictive factors for severity of prematurity risk in our cohort of prematurely delivered dichorionic twin gestations after assisted reproduction. Since our study population consisted of a limited number of good prognosis patients, our results do not preclude that further research in more typical average IVF populations, at larger risk for prematurity, may after all, be able to define such risk factors. Further research is, therefore, warranted to develop a risk-focused approach to prevent, or at least reduce, adverse perinatal outcomes in twin pregnancies after ART.

## Competing interests

The authors declare that they have no competing interests.

## Authors' contributions

AW Substantial contributions to conception and design, acquisition, analysis and interpretation of data, drafting the article, final approval of the version to be published. KK and MSS Substantial contributions to acquisition and contributions to analysis of data, revising the article critically for important intellectual content, final approval of the version to be published. DHB, CW, PH Substantial contributions to conception of the study, analysis of data, revising the manuscript critically for important intellectual content, final approval of the version to be published. NG Substantial contributions to conception and design, analysis and interpretation of data, revising the manuscript critically for important intellectual content, and final approval of the version to be published.
